# Officiating stress and coping strategies among male student basketball referees in China: a procedural grounded theory study

**DOI:** 10.3389/fpsyg.2026.1794393

**Published:** 2026-06-26

**Authors:** Jinchen Pan, Shuo Fang, Ziyuan Quan, Xin Huang, Lei Zheng

**Affiliations:** 1 China Basketball College, Beijing Sport University, Beijing, China; 2 School of Physical Education, Soochow University, Suzhou, Jiangsu, China; 3 School of Psychology, Beijing Sport University, Beijing, China

**Keywords:** decision-making pressure, male student basketball referees, officiating stress, procedural grounded theory, psychological resilience, stress coping strategies

## Abstract

**Introduction:**

This study aimed to explore the sources, interactions, outcomes, and coping strategies associated with officiating stress among male student basketball referees in China.

**Methods:**

Using a procedural grounded theory approach, 28 male student basketball referees participated in semi-structured, in-depth interviews. Purposive sampling was initially employed to ensure sample diversity, followed by theoretical sampling based on constant comparison. Data were analyzed using open, axial, and selective coding with the assistance of NVivo 12.0.

**Results:**

A total of 979 coding references were identified, generating 53 initial categories, 15 subcategories, and five main categories: personal factors, relational pressure, decision-making pressure, match pressure, and event-related pressure. These dimensions formed an interactive, multifactor model of officiating stress. Theoretical saturation was reached after the 26th interview, and two additional interviews confirmed that no new categories or relationships emerged. Moderate pressure could facilitate learning and professional growth, whereas excessive pressure could contribute to stress responses, officiating errors, and a self-reinforcing cycle of escalating pressure.

**Discussion:**

Coping strategies included self-regulation, social support, skill development, and stress-response adjustment, although organizational support remained insufficient. The findings provide practical implications for developing psychological support systems, structured training pathways, post-match review mechanisms, and referee development programs.

## 1 Introduction

Referees play a crucial role in maintaining fairness, order, and procedural legitimacy in competitive sport. In basketball, referees are responsible for overseeing the game, enforcing rules, interpreting players’ actions, and making rapid decisions under conditions of intense physical contact, time pressure, uncertainty, and spectator pressure (Chen, 2020; International Basketball Federation, 2024). Their decisions may directly influence the flow and outcome of the game, as well as the fairness perceived by players, coaches, spectators, and other officials (Qu, 2012; Wang and Wang, 2005). Because officiating requires sustained attention, rapid information processing, emotional regulation, and continuous interaction with players, coaches, spectators, and other officials, referees are often exposed to multiple psychological stressors during competition.

International research has provided important evidence that officiating stress represents a meaningful psychological issue across different sports and competitive contexts. Early studies identified acute stressors among basketball referees and examined how personal and situational factors predicted coping strategies under pressure (Anshel and Weinberg, 1995; Kaissidis-Rodafinos et al., 1997). Research on baseball, softball, and rugby referees further showed that officiating stress may be associated with performance concerns, criticism from coaches and spectators, burnout, and even the intention to discontinue refereeing (Rainey, 1995; Rainey and Hardy, 1999). More recent studies have extended this discussion to different sports and officiating environments, indicating that referee stress may vary according to role, competition level, contextual demands, emotional regulation, and mental well-being (Karaçam et al., 2023; Nikolovski et al., 2024; Suárez-Iglesias et al., 2021). In addition, studies on refereeing performance have highlighted the importance of decision-making skills, teamwork among officials, rule interpretation, and communication in shaping officiating quality and stress-related experiences (MacMahon et al., 2007; Sabag et al., 2023).

Although these studies have advanced our understanding of officiating stress, several issues remain underexplored. First, much of the existing literature has focused on identifying specific stressors or examining associations between stress and psychological or performance-related variables. Such research is valuable for describing general patterns, but it is less able to explain how different sources of pressure interact during the officiating process, how referees interpret stress in specific match contexts, and how stress experiences become linked to coping strategies over time. Second, existing studies have paid greater attention to professional referees, elite referees, or referees as a broad occupational group, whereas student referees in collegiate competition have received comparatively limited attention. Student referees occupy a transitional developmental stage: they are still accumulating practical experience, building professional confidence, adapting to evaluation and promotion systems, and learning to manage complex interpersonal interactions during competition. Their stress experiences may therefore differ from those of more experienced professional referees.

The Chinese collegiate basketball context provides a particularly important setting for examining these issues. With the rapid development of basketball in Chinese universities and the increasing demand for referee talent, university students have been described as an important source of basketball referees (Wang, 2016). Universities provide formal courses, referee training opportunities, and practical officiating platforms, while national and student-level competitions expose young referees to increasingly demanding match environments (Pei, 2002). The Chinese Basketball Association has also emphasized the training and long-term development of young referees, further highlighting the practical importance of this group (China Sports Daily, 2020). Male student basketball referees who have obtained National Level-I certification are especially representative because they possess a recognized qualification standard but often remain in a developmental stage of professional growth. They may face pressure related to limited high-level officiating experience, decision-making accuracy, peer coordination, promotion expectations, organizational evaluation, and participation in national-level competitions. However, the sources of officiating stress and the ways in which male student basketball referees cope with such stress in the Chinese collegiate context remain insufficiently understood.

To address this gap, the present study adopted a procedural grounded theory approach to explore officiating stress and coping strategies among male student basketball referees in China. Rather than testing a predetermined theoretical model, this study aimed to develop a process-oriented and context-sensitive understanding of how these referees perceive, interpret, and cope with pressure during officiating. Specifically, the study addressed the following research questions:

RQ1: What are the main sources of officiating stress experienced by male student basketball referees in China?

RQ2: How do different types of officiating stress interact to shape referees’ psychological responses and perceived officiating performance during competition?

RQ3: What coping strategies do male student basketball referees use to regulate and manage officiating stress?

By answering these questions, this study sought to construct an empirically grounded model of officiating stress and coping among male student basketball referees. The findings may contribute to the literature on referee psychology by offering an integrative and process-oriented account of stress sources, stress appraisal, and coping strategies in the Chinese collegiate basketball context. From a practical perspective, this study may also provide implications for referee training, psychological support, post-match evaluation mechanisms, and developmental pathways for young basketball officials.

## 2 Methodology

### 2.1 Research approach

This study investigates the exploratory topic of refereeing pressure experienced by university basketball referees. As a reality-oriented rather than theory-testing inquiry, the complexity and contextual variability of referees’ pressures and coping processes make a grounded theory approach particularly appropriate. Grounded Theory (GT), originally developed by Glaser and Strauss (Glaser and Strauss, 1967), has evolved into three main strands: classical, procedural, and constructivist grounded theory. After comparing these approaches, this study adopts procedural grounded theory as the primary methodological framework. The analytical process follows the sequential logic of open coding, axial coding, and selective coding.

While procedural grounded theory serves as the core framework, this study also draws selectively on compatible methodological principles from other GT traditions to enhance analytical rigor. From classical grounded theory, we adopt constant comparison, theoretical sensitivity, theoretical sampling, and theoretical saturation. From constructivist grounded theory, we incorporate reflexive awareness and contextual interpretation, recognizing that categories are constructed through the interaction between researchers, participants, and the research context. Accordingly, this study is grounded primarily in procedural grounded theory, while being informed by a reflexive and context-sensitive interpretive stance.

Procedural grounded theory emphasizes continuous comparison and iterative abstraction, enabling the development of conceptual categories and theoretical explanations directly from empirical data. This approach is well suited to examining the mechanisms underlying officiating pressure and coping strategies among university basketball referees. To avoid methodological formalism, this study followed the principles of theoretical sensitivity, constant comparison, and iterative category refinement, with reflexivity maintained throughout data collection and analysis (Shi and Huo, 2021; Chen and Jiang, 2025; Lin et al., 2024).

### 2.2 Data collection

This study adopted a sampling strategy consistent with procedural grounded theory. During the iterative process of data collection and analysis, the research team continuously adjusted participant selection and interview focus in response to the development of emerging concepts and categories. No fixed sample size was predetermined before data collection. Interviews were terminated only when no substantially new concepts or categories emerged during open, axial, and selective coding, and when the relationships among the core categories had become sufficiently stable. Before the interviews, all participants were informed of the study purpose, research procedures, voluntary nature of participation, and confidentiality arrangements. Written informed consent was obtained from all participants. This study was approved by the Ethics Committee of Beijing Sport University (Approval No. 2025305H) as Supplementary Document 3. This study was reported in accordance with the Standards for Reporting Qualitative Research (SRQR) (O'Brien et al., 2014), and the completed checklist is provided as Supplementary Document 4.

The interviews were conducted by an experienced National Level-I Basketball Referee certified by the Chinese Basketball Association, who was in the process of promotion to the national-level referee qualification. This professional background enabled the interviewer to understand the technical terminology, competitive context, and practical challenges of basketball officiating. In addition, one member of the research team was an international-level basketball referee with regular experience officiating world-level competitions. This expertise provided important support for understanding the professional context of refereeing, refining the interview questions, and interpreting domain-specific terminology during data analysis.

At the same time, the research team recognized that such insider knowledge might introduce interpretive bias. To reduce this risk, the interviewer used a semi-structured interview format with open-ended questions, avoided leading or evaluative responses, and encouraged participants to describe their experiences in their own words. During data analysis, reflexive memos were maintained, and the research team repeatedly returned to the original interview transcripts to ensure that interpretations remained grounded in participants’ accounts rather than in the researchers’ prior assumptions.

Potential participants were contacted through the Referee Association of Beijing Sport University. In the initial recruitment stage, 37 individuals were invited to participate in the study; 34 agreed to be interviewed, 1 declined, and 2 did not respond, resulting in a response rate of 92%. Among those who agreed to participate, 28 ultimately completed the interviews. Of these 28 participants, 15 were known to the research team through prior competition-related experience, whereas the remaining 13 were contacted through referee training groups. All potential participants were formally approached only after the study purpose, voluntary nature of participation, and confidentiality arrangements had been clearly explained. No participant had a direct dependent, subordinate, or evaluative relationship with the interviewer.

All participants in the final analytic sample were student referees from universities in China and had officiated national-level competitions, including the Chinese Youth Basketball League and national student basketball competitions. All participants were certified National Level-I Basketball Referees recognized by the Chinese Basketball Association, ensuring a comparable level of officiating qualification and professional experience within the sample. The inclusion of only male National Level-I student referees was intentional, as it reduced variation attributable to both gender and certification level and allowed the study to focus more specifically on differences in officiating experiences, stress perceptions, and coping processes among referees operating within a comparable qualification framework. In total, 28 male student basketball referees were included in the final analysis, with a mean age of 23.43 ± 2.33 years and a mean officiating experience of 4.29 ± 2.14 years (Participant characteristics are presented in Supplementary Document 5).

All interviews were conducted in Mandarin Chinese, either face to face or via Tencent Meeting. The mean interview duration was 47 min, with individual interviews lasting approximately 30 to 63 min. All interviews were audio-recorded with participants’ consent. This study adopted a semi-structured interview format, in which the interviewer used open-ended questions, avoided leading responses, and allowed participants to describe their officiating experiences in their own words. The interview guide focused on concrete officiating experiences, perceived stress, sources of stress, stress-related outcomes, coping strategies, and personal reflection. Because the participants constituted a specific and information-rich group with direct experience in collegiate basketball officiating, they were able to provide focused and experience-based accounts of officiating stress and coping processes. The complete interview guide is provided as Supplementary Document 1 to enhance methodological transparency and allow readers to evaluate the scope and focus of the interviews.

Data collection was conducted in two phases. The initial phase took place from January 17 to May 13, 2025. During this phase, interviews and preliminary analysis proceeded iteratively, with emerging concepts and category development informing subsequent participant selection and adjustments to the focus of later interviews. Following refinement of the study scope to focus exclusively on male student basketball referees, two additional eligible male referees were recruited and interviewed on May 21 and 22, 2026, as confirmatory cases. The same eligibility criteria, semi-structured interview guide, recording procedures, and analytic approach were applied in both phases. The two additional interviews were incorporated into the constant comparative analysis and did not generate any new categories, category properties, or inter-category relationships.

The transactional model of stress and coping was used only as a sensitizing framework to inform the design of broad interview questions concerning stress perception, appraisal, and coping. It did not serve as a predetermined coding framework (Lazarus and Folkman, 1984). The analysis remained grounded in participants’ accounts, and categories were generated inductively through constant comparison. In other words, the theory helped guide the development of open-ended interview prompts, whereas the identification of categories and their relationships was based on the empirical interview data.

Theoretical sampling was guided by three criteria: first, when a concept recurred but its properties and boundaries remained unclear; second, when overlapping categories required further differentiation; and third, when the relationships between categories and the emerging core category remained unstable.

Theoretical saturation was assessed at three levels: conceptual saturation, categorical saturation, and relational saturation. At the conceptual level, each new interview was compared with previously analyzed data to determine whether new concepts emerged. At the categorical level, constant comparison was used to assess whether the properties and dimensions of each category had been sufficiently developed. At the relational level, the stability of relationships among the core categories was examined.

Theoretical saturation was considered to have been reached after the 26th male interview. At that point, no substantially new concepts emerged, the properties of the main categories had become repetitive and well developed, and the relationships among decision-making pressure, match pressure, relational pressure, and personal factors had become stable across cases. Two additional male interviews were subsequently conducted as confirmation cases. These two interviews did not generate new categories, new category properties, or new inter-category relationships. Therefore, sampling was terminated based on conceptual redundancy, category stability, and the stability of the emerging theoretical model. To further support this judgment, Supplementary Table 2 presents the trajectory of concept and category emergence across the 28 male interviews.

### 2.3 Interview text mining analysis

NVivo 12.0 was used as an auxiliary tool for data organization, coding, retrieval, and comparison; however, analytic interpretation was conducted independently by the research team and was not software driven. Data analysis followed the three-stage coding procedure of procedural grounded theory, including open coding, axial coding, and selective coding.

All transcripts were de-identified before analysis. Participants were assigned anonymized identification codes (R01–R28), and names, institutional affiliations, competition-specific details, and other potentially identifying information were removed or generalized in the transcripts and reported quotations.

Throughout the analytic process, the research team applied constant comparison across all interview data. Each newly coded transcript was compared with previously analyzed data to determine whether new concepts had emerged, whether existing categories required refinement, and whether the relationships among categories remained stable.

During open coding, initial concepts were generated line by line from participants’ narratives. During axial coding, related concepts were grouped and reorganized into higher-order categories based on conceptual similarity and contextual relationships. During selective coding, the core categories were further integrated into an overarching storyline, leading to the development of a theoretical model of officiating stress among male student basketball referees.

Data analysis was conducted by three members of the research team. The first coder had training in qualitative research and experience in basketball officiating; the second coder had a background in sport psychology and qualitative data analysis; and the third researcher served as an adjudicator during category refinement and theoretical integration. In the initial stage, two coders independently conducted line-by-line open coding on a subset of transcripts and generated preliminary codes. The coding results were then compared in team meetings to refine code names, clarify category boundaries, and develop shared coding definitions.

When coding discrepancies occurred, the researchers first returned to the original Chinese transcripts and examined the surrounding context of participants’ statements. Disagreements were resolved through discussion until consensus was reached. If the two coders could not reach consensus, the third researcher reviewed the relevant transcript segments and participated in the final decision. After the preliminary coding framework had been refined, the remaining transcripts were coded through iterative comparison, with new codes and category modifications discussed collectively by the research team.

Formal inter-coder reliability statistics were not calculated because this study followed a procedural grounded theory approach, in which coding is regarded as an interpretive and comparative process for category development rather than as a fixed coding scheme for quantitative reliability testing. Nevertheless, analytic dependability was strengthened through independent preliminary coding, repeated team discussions, consensus-based resolution of discrepancies, memo writing, constant comparison, and the maintenance of an audit trail documenting coding decisions, category refinement, theoretical sampling, and saturation assessment.

Formal inter-coder reliability statistics were not calculated because this study followed a procedural grounded theory approach, in which coding is regarded as an interpretive and comparative process for category development. To enhance analytic dependability, the research team conducted independent preliminary coding, held repeated team discussions, resolved discrepancies through consensus, maintained analytic memos, applied constant comparison, and established an audit trail documenting coding decisions, category refinement, theoretical sampling, and saturation assessment. To enhance the credibility of this research, the research team adopted several strategies. Through real-time clarification during the interview process, member verification of some participants after the initial category formation, continuous comparison during data analysis, and the use of representative quotations to support the analysis and explanations, the credibility of the research was strengthened.

## 3 Results

### 3.1 Open coding

A total of 979 meaning units were identified and coded in this study. Through multiple rounds of inductive analysis and constant comparison, 53 initial categories were generated. All extracted concepts were related to refereeing pressure experienced by university basketball referees (Table 1). By further examining the properties and semantic relationships of these concepts, similar codes were grouped and integrated based on conceptual similarity and logical connections, resulting in 15 higher-order categories.

**Table 1:** Examples of open coding and initial categories.

Initial category	Concept	Original text
physiological load	Declining physical fitness and impaired concentration	I need to provide more details about my matches, particularly the impact on concentration following a decline in physical fitness.(R02, 4 years of experience)
self-efficacy	Self-doubt breeds internal anxiety.	During the match, if I feel I’ve made a mistake in my officiating, I become extremely anxious even without any external pressure.(R07, 3 years of experience)
Pressure from differences in experience	The presence of senior referees officiating the same match creates pressure.	The presence of senior referees officiating at the same venue has created pressure.(R09, 2 years of experience)
Pressure to maintain consistent standards in officiating	Key officiating decisions must maintain consistency in standards.	When making the second call—that is, in a 50–50 situation—if I make the call, if I make that critical call, I must pay close attention to similar situations arising in subsequent plays and ensure I make the correct judgment every time.(R10, 10 years of experience)
Multiple pressures compound on the field	High-intensity matches and the spectator atmosphere collectively increase pressure on referees.	First, the crowd was huge for that game. Second, the match was incredibly intense, going to two tiebreaks before a winner emerged. Third, both players showcased exceptional technical skills, resulting in fierce competition. Physical contact on the court was frequent, leading to numerous fouls.(R25, 4 years of experience)

### 3.2 Core axis coding

Based on open coding results, axial coding further examined the relationships among categories and their underlying conceptual structures. Through iterative comparison and integration, the 15 subcategories were systematically organized into five main categories. These five categories form the core structure of refereeing pressure among university basketball referees (see Table 2).

**Table 2:** Axial coding results.

Primary category	Corresponding category	Includes the concept
Personal factors	Physical factors	Action execution, physiological load, mental fatigue
	Psychological factors	Emotional regulation ability, psychological sensitivity, psychological resilience, self-efficacy
	Professional competence	Individual officiating competence, communication and coordination skills, mastery of rules, on-the-spot adaptability and decision-making ability, and accumulated officiating experience.
Relationship stress	Interpersonal stress	Interpersonal pressure among referees, interpersonal interaction pressure within the organizing committee
	Social pressure	Media coverage, peer reviews, online public opinion
	Pressure on referee coordination	Differences in experience pressure, lack of coordination, and variations in officiating styles
	Organizational relationship pressure	Evaluation scoring pressure, promotion pressure, seasonal task pressure, insufficient organizational support pressure
Pressure of refereeing decisions	Pressure on referee decision-making	Conflict between fairness and subjective judgment in officiating, pressure to maintain consistent standards in officiating
	Pressure of referee decisions	The intensity of the game’s pace, the comprehensive adjudication of multi-factor conflict scenarios, the complexity and stealthiness of foul actions, and the disconnect between rule comprehension and on-field situations.
	Timing of penalty calls	Ruling too hastily: Rash, erroneous judgment Ruling too slowly: Missing the opportunity
	Accuracy of refereeing decisions	Accuracy of critical calls, pressure from missed or incorrect calls, and accurate calls boosting confidence.
Competition pressure	Competition environment pressure	Venue environment induces stress, multiple pressures compounding on the field, unexpected situations during play, spectator atmosphere exerting pressure, coaching staff applying pressure, athletes pressuring themselves.
	Competitive pressure on the field	Competition pressure, match intensity, scoreboard pressure, critical calls, player skill level
Competition and match pressure	Crucial matches	Championship showdown, powerhouse clash, rivalry showdown, knockout stage advancement, home team’s crucial match
	Competition level	The heightened standards for high-level competitions create pressure, and the outcomes of such competitions carry significant implications.

### 3.3 Selective coding

**Figure 1:**
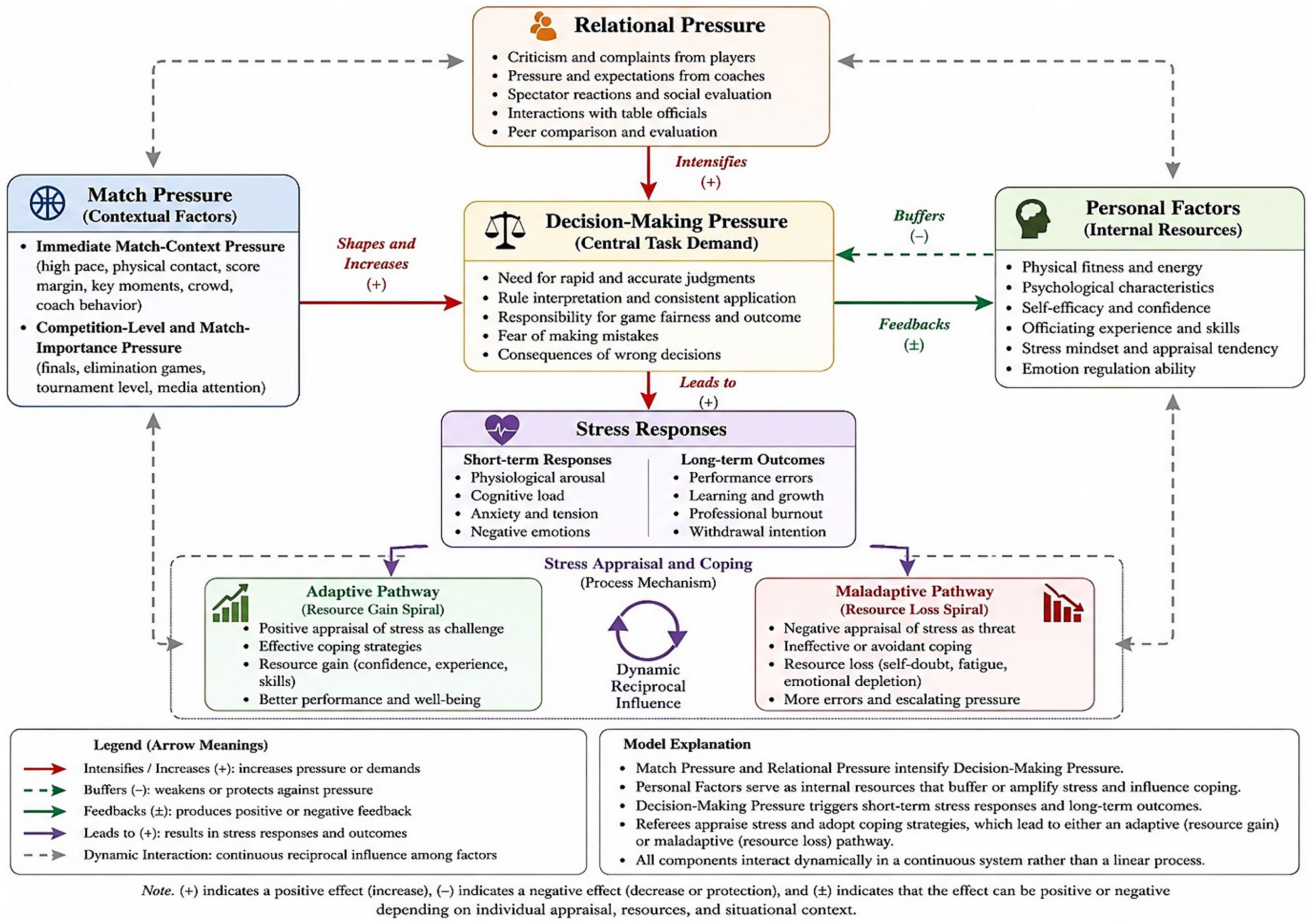
An integrated model of officiating stress in college basketball referees.

Building upon axial coding, this study conducted selective coding around five core categories: personal factors, decision-making pressure, relational pressure, match pressure, and event-related pressure. These categories were further integrated through an emergent storyline, revealing the structural relationships among the core dimensions. This process led to the development of the College Basketball Refereeing Pressure Model (Figure 1). No new major concepts emerged after the 26th interview. More importantly, the properties and dimensions of the five core categories became stable and well developed, and the relationships among categories remained consistent across subsequent comparisons. To further assess theoretical saturation, two additional interviews were conducted as verification cases. These interviews did not yield any new category properties, dimensions, or inter-category relationships, thereby supporting the conclusion that theoretical saturation had been achieved.

### 3.4 Analysis of refereeing pressure among college basketball officials

#### 3.4.1 Personal factors: the internal foundation for stress coping

Personal factors include physical fitness, psychological characteristics, and professional competence, forming the internal resource system through which referees respond to external demands. “After playing the entire match, my legs became completely weak; in the final minutes, I simply could not keep up with the ball’s speed, leading to frequent misjudgments.(R24, 2 years of experience)” Existing evidence on basketball game workload suggests that high-level performance requires greater adaptation to intensity rather than volume of load. At the refereeing level, rapid offensive–defensive transitions place high demands on both physical capacity and sustained attention, which helps explain why fatigue may lead to delayed judgment and emotional instability during prolonged officiating. Psychological characteristics such as anxiety and emotional sensitivity may reduce decision confidence and response stability, whereas professional competence directly influences rule application, situational interpretation, and decision accuracy. “Psychological resilience is crucial for referees. An outstanding referee must quickly regain composure after making a controversial call and avoid allowing emotions to interfere with subsequent officiating decisions, which requires long-term, systematic psychological training.(R03, 4 years of experience)” From a stress appraisal perspective, individual resource levels shape how referees evaluate match demands. Referees with limited rule knowledge, insufficient experience, or lower psychological resilience are more likely to appraise high-intensity situations (e.g., close scores, physical contact, coaching pressure) as exceeding their coping capacity, leading to heightened stress responses. In contrast, referees with stronger competence and experience are more likely to interpret similar situations as manageable challenges, resulting in more stable stress responses. At the response level, personal factors further influence cognitive efficiency, emotional regulation, and behavioral execution under pressure. “The accumulation of refereeing experience is irreplaceable. While young referees often demonstrate precise technical skills, their ability to make comprehensive judgments in chaotic situations and their communication skills with athletes and coaches require time and extensive practical experience to develop.(R10, 10 years of experience)” Physical fatigue reduces attentional control and positioning quality, particularly in later stages of matches. Lower psychological resilience may lead to hesitation or reduced decisiveness when facing controversial calls or perceived errors, whereas higher competence and self-regulation ability facilitate faster recovery and more stable officiating performance. Overall, personal factors play a decisive role in shaping whether referees follow a stress–error–escalation or a stress–adjustment–stabilization pathway. Even under identical match conditions, referees with stronger internal resources tend to maintain higher stability in perception and decision-making, whereas those with weaker resources are more likely to experience intensified stress reactions and performance fluctuations.

#### 3.4.2 Penalty pressure: the core stress of refereeing

Decision-making pressure represents the most direct and prominent form of refereeing pressure and includes four sub-dimensions: standard consistency, task difficulty, timing demands, and decision accuracy. These dimensions reflect the core cognitive demands placed on referees during officiating tasks. A fundamental tension underlies this pressure: as one experienced referee observed, “A persistent tension exists between the fairness of officiating and the inherent cognitive limitations of referees. How to achieve the greatest possible objectivity and fairness, given the inevitability of subjective judgment, remains a lifelong challenge for every referee” (R10, 10 years of experience). In fast-paced and high-intensity games, overlapping situational factors, complex contact situations, and ambiguous rule application further increase the difficulty of maintaining consistent and accurate decisions. This challenge is compounded by the evolving nature of the game itself: as one referee reflected, “Modern basketball’s offensive and defensive intensity, along with its tactical complexity, far exceed the scenarios originally envisioned when the rules were established. Many real-world conflict situations cannot be precisely covered by the rulebook, requiring referees to exercise holistic judgment that transcends legal provisions” (R23, 7 years of experience). Timing demands require referees to make rapid judgments; premature calls may lead to errors, whereas delayed decisions may result in missed calls, both of which increase psychological strain. The cost of hesitation in such moments was described pointedly by one participant: “Hesitation is a major pitfall. I’ve witnessed referees taking two seconds to make a call, by which time the players had already engaged in combat, exacerbating the chaos on the field. Once the critical moment is missed, even the most accurate decision appears ineffective” (R05, 6 years of experience). Accuracy-related pressure reflects not only the risk of incorrect decisions but also the stabilizing effect of correct judgments on confidence: “Recurrent misjudgments erode confidence and foster refereeing anxiety, whereas high-quality accurate calls provide positive reinforcement, helping referees establish a stable psychological foundation for officiating” (R21, 8 years of experience). The continued evolution of basketball rules and match situations has also increased the complexity of on-court decision-making for referees (Lu, 2011; Li, 2014).

From a structural perspective, match pressure and decision-making pressure operate at different levels. Match pressure refers to situational demands arising from the competitive environment, including crowd atmosphere, coaching behavior, game tempo, physical intensity, score fluctuations, and critical moments. Decision-making pressure, in contrast, refers to the cognitive and psychological demands involved in executing officiating judgments. Match pressure functions as an antecedent condition that intensifies decision-making demands. More complex and high-intensity match contexts increase information load and reduce decision time, thereby elevating decision-making pressure. In this sense, match pressure is external and contextual, whereas decision-making pressure is task-specific and performance-oriented. Together, they form a hierarchical relationship in which contextual demands translate into cognitive–decisional strain during officiating tasks.

#### 3.4.3 Relational stress: interference from interpersonal relationships

Relational stress consists of four subcategories: routine interpersonal interactions, referee coordination, public evaluation, and organizational pressures. These dimensions reflect stressors arising from both internal team dynamics and external evaluative environments.

College basketball referees reported challenges related to differences in experience levels among officiating partners, insufficient coordination, and variation in officiating styles. The interpersonal dimension of these challenges extends beyond purely professional disagreements, as one referee noted: “There exists an underlying interpersonal tension within the refereeing team, where seniority, factions, and personal interests all influence relationships among members. These external factors can sometimes manifest on the field, affecting the referees’ performance” (R18, 3 years of experience). At the level of on-court coordination specifically, participants emphasized that structural and stylistic misalignment compounds these relational difficulties: “The overall coordination of the refereeing team is crucial to ensuring high-quality officiating; differences in style, experience gaps, or inadequate communication mechanisms can lead to inconsistent penalty standards, necessitating thorough collaboration before the match” (R16, 4 years of experience). In addition, external pressures from spectators, media commentary, and online public opinion further contribute to relational stress. The asymmetric nature of media attention was a recurring concern among participants: “Media coverage of referees is often selective; any controversial call is invariably amplified. Under prolonged public scrutiny, referees’ professional dignity suffers significant erosion” (R25, 4 years of experience). At the organizational level, stressors such as evaluation systems, promotion competition, season-long task requirements, and perceived lack of institutional support also emerged as important sources of pressure. The chronic nature of this strain was captured by one referee’s account of promotional competition: “There are only a limited number of promotion spots, and everyone is competing fiercely. You know someone is watching every single one of your performances—this pressure lasts longer than that on the field itself” (R13, 3 years of experience). These factors reflect a more sustained form of stress embedded within the organizational context of refereeing work. Overall, relational stress represents a multi-layered pressure system that includes interpersonal, evaluative, and organizational dimensions, all of which interact to influence referees’ perceived workload and psychological experience during officiating.

#### 3.4.4 Competition pressure: situational stress in the game environment

Competition pressure refers to situational stressors arising from both the environmental context and competitive dynamics of the game. At the environmental level, referees reported pressure induced by venue conditions, including crowd presence, auditory and visual distractions, and the overall competitive atmosphere created by spectators, coaches, and players. The cumulative weight of these simultaneous stimuli was described by one participant: “Lighting conditions, noise levels, audience emotions, coaching interventions, and athlete pressure collectively create a highly stressful refereeing environment. Referees must possess the ability to maintain focus and composure amidst these multiple stimuli” (R22, 5 years of experience). These factors require sustained attentional control under complex sensory conditions. At the competitive level, match progression and game tempo contribute to pressure experiences. Score fluctuations, critical game moments, and differences in player performance levels further increase the cognitive demands associated with decision-making. This demand is particularly pronounced when officiating high-caliber players, as one referee reflected: “When facing top-tier teams, the pace of play is extremely fast and physicality intense; some technical fouls are concealed within seamless teamwork and hard to spot. The more skilled the players, the greater the challenge for referees—only those who truly understand the game can effectively manage them” (R23, 7 years of experience). Overall, competition pressure reflects the combined influence of environmental stimuli and competitive dynamics, which continuously shape referees’ perceived situational demands during officiating.

#### 3.4.5 Tournament and match pressure: structural pressure from tournament scheduling

Tournament and match pressure refers to stressors arising from both critical game contexts and competition level. At the match level, high-stakes games—such as championship finals, rivalry matches, knockout-stage games, and decisive fixtures—are associated with increased attention and evaluative scrutiny, which elevate referees’ perceived situational demands. The convergence of multiple sources of scrutiny in such contexts was vividly captured by one participant: “The refereeing pressure in finals or high-stakes matches is unique: attention from both teams, club management, media, and fans reaches its peak simultaneously, requiring referees to handle exceptionally demanding technical and tactical challenges” (R16, 4 years of experience). At the tournament level, higher-tier competitions typically involve stricter officiating standards, increased media exposure, and greater consequences associated with match outcomes. These conditions contribute to elevated cognitive and decision-making demands for referees, a reality reflected in one referee’s account of the asymmetric visibility of errors across competition tiers: “The higher the competition level, the lower the fault tolerance rate. A similar missed call might go unnoticed in lower-tier events, but at national-level competitions, it appears in various analysis videos the very next day” (R4, 7 years of experience). Overall, tournament and match pressure reflects structurally embedded demands associated with game importance and competition level, which jointly shape referees’ perceived officiating load.

#### 3.4.6 Structural relationships among stress factors

This study identified an integrated structural relationship among the five dimensions of refereeing pressure among college basketball referees. Field pressure, tournament and match pressure, and relational stress jointly constitute external situational demands that influence referees’ decision-making pressure during officiating tasks. Decision-making pressure functions as the central dimension of the model, reflecting cognitive demands related to judgment consistency, difficulty, timing, and accuracy. Personal factors operate as internal resources that shape referees’ capacity to respond to external demands, influencing how situational pressures are perceived and managed. Overall, the findings suggest a layered structure in which external pressures (field, tournament, and relational contexts) are translated into decision-making pressure, while personal factors modulate the extent to which these pressures are experienced during officiating. The relationships among these dimensions form the overall analytical framework of refereeing pressure in collegiate basketball contexts.

## 4 Discussion

The model of officiating pressure among college basketball referees proposed in this study is theoretically consistent with the transactional model of stress and coping (Lazarus and Folkman, 1984). Similar stress patterns have also been observed among referees in other sports, suggesting that officiating pressure may share common underlying mechanisms across different sport contexts. This further supports the potential generalizability of the present model beyond basketball-specific settings (Nikolovski et al., 2024). The transactional model emphasizes that stress does not arise solely from environmental stimuli. Instead, it results from the interaction between the individual and the environment through cognitive appraisal and coping. Cognitive appraisal includes primary appraisal, which concerns whether a stressor is perceived, and secondary appraisal, which concerns whether one’s coping resources are sufficient. The pressure model developed in this study reflects a similar logical structure. In this model, match pressure acts as a contextual antecedent that shapes decision-making pressure. Specifically, the multidimensional stressors faced by referees, including match pressure, decision-making pressure, and relational pressure, constitute the situational inputs. Personal factors, such as physical fitness, psychological state, and professional competence, influence how referees appraise these stressors. Coping strategies then determine whether pressure develops into a growth cycle or a loss cycle. The main difference is that the model proposed in this study further reveals the dynamic cyclicality of stress appraisal and coping. Successful coping can strengthen personal resources and shift pressure perception toward challenge, whereas unsuccessful coping may deplete personal resources and intensify the negative effects of pressure. In this sense, the present model complements the transactional model by explaining the cumulative and cyclical process through which officiating pressure develops over time.

### 4.1 Analysis of stress sources for college basketball referees

The stress experienced by college basketball referees is multidimensional and interrelated. Based on qualitative analysis of semi-structured interviews, this study identifies four core dimensions of officiating pressure among college basketball referees: personal factors, relational pressure, decision-making pressure, and match pressure. These dimensions reflect stress sources that are particularly relevant to student basketball referees during their professional development. (1) Personal factors: Personal factors represent an important source of pressure. College basketball referees are typically at a developmental stage of professional growth, during which their physical fitness, psychological resilience, and officiating competence are still developing. Research indicates that individuals with low self-efficacy often lack confidence, fear making mistakes, and worry about failing to complete tasks (Bandura, 1997). Empirical research also suggests that age may moderate the relationship between coping styles and emotional states among basketball referees (Wang et al., 2023). Inadequate professional competence may directly exacerbate anxiety during matches. For example, incomplete rule knowledge may lead to hesitation in high-intensity situations and contribute to reduced confidence and self-blame (Xin et al., 2012; Ye and Chen, 2016). External pressures, including match importance and physical intensity, may amplify perceived capability gaps and contribute to a cycle of skill deficiency, hesitation, and heightened stress (Jamieson et al., 2010; MacMahon et al., 2007). Basketball refereeing also imposes substantial physical demands; referees may cover approximately 4,000–6,000 m per game (Nabli et al., 2016). They must maintain appropriate positioning, monitor designated areas, and make accurate decisions throughout the match (International Basketball Federation, 2024). Fatigue may reduce concentration and decision accuracy, particularly during the later stages of competition (Nabli et al., 2016). (2) Relational pressure: Relational pressure arises from interactions with other officials, players, coaches, spectators, and the organizational environment. These interactions may affect referees’ perceived fairness, confidence, and psychological stability (Xing and Zhao, 2021). Team-related pressures may be particularly salient among less experienced referees (Weinberg and Richardson, 1990). Trust and coordination among colleagues can provide psychological support and facilitate joint decision-making in complex situations (Yu and Pan, 2010; Sabag et al., 2023). (3) Decision-making pressure: Decision-making pressure is central to basketball officiating and concerns the consistency, complexity, timing, accuracy, and acceptance of decisions. Less experienced referees may hesitate because they fear being perceived as either too lenient or too strict. (4) Match pressure: Match pressure acts as a contextual antecedent that shapes decision-making pressure. It arises from match pace, physical intensity, importance, competition level, and critical moments. High-level tournaments and closely contested matches increase task complexity and external scrutiny, thereby intensifying decision-making pressure (Suárez-Iglesias et al., 2021; Goldsmith and Williams, 1992).

### 4.2 Analysis of stress coping strategies among college basketball referees

College basketball referees adopt diverse coping strategies when facing complex and multifaceted stressors. However, the effectiveness and applicability of these strategies vary across individuals and situations.

Self-regulation strategies are commonly used by college basketball referees to manage immediate stress responses. Positive self-talk, pre-match mental preparation, and breathing-based relaxation may help referees regulate short-term anxiety and enhance perceived readiness (Voight, 2009). However, such short-term techniques do not fully address deeper stressors, including insufficient professional competence or complex interpersonal dynamics. Mindfulness training has received increasing attention as a more systematic self-regulation method. Research suggests that mindfulness training may help college student basketball referees improve concentration, reduce rumination, and lower on-court anxiety (He, 2024). By cultivating awareness and acceptance of present experiences, mindfulness may strengthen psychological resilience and help referees maintain composure under pressure. Nevertheless, its benefits generally require sustained practice and appropriate professional guidance.

Social support strategies serve as an important external resource for college basketball referees. Stronger social support is generally associated with greater psychological benefits (Li, 1998). Emotional support and coordination among peers may alleviate anxiety and feelings of isolation during high-pressure matches. Team trust has also been associated with cooperative performance and cohesion in competitive sport (Yu and Pan, 2010). During officiating, eye contact, coordinated gestures, and real-time communication can help officials manage complex match situations collectively. Effective teamwork and coordination among officials may improve decision quality and strengthen confidence during officiating (Sabag et al., 2023; Dong et al., 2021). However, formal organizational support remains limited, and many universities and sport organizations have not established routine psychological consultation, structured mentoring, or systematic experience-sharing mechanisms for referees.

Strategies for skill enhancement are fundamental to strengthening referees’ capacity to cope with stress. Continuous study of basketball rules, rule interpretations, and referee manuals provides a theoretical foundation, while extensive match experience supports practical understanding. Post-match video review, simulation-based practice, and expert feedback may help referees identify decision-making errors and refine officiating performance (MacMahon et al., 2007; Voight, 2009). Simulation training also allows referees to rehearse high-pressure situations in a controlled environment. Adequate physical preparation is essential for maintaining positioning, attentional control, and decision-making performance throughout a match (Nabli et al., 2016). Nevertheless, skill development requires sustained time and access to appropriate training resources, which may be constrained by student referees’ academic commitments.

The coping strategies identified in this study mainly focus on individual adjustment and proximal social support, including self-regulation, peer support, rule learning, post-match review, and simulation training. This reflects the current reality that college basketball referees mainly rely on personal resources and informal support networks when facing pressure. However, this does not mean that organizational-level coping is unimportant. On the contrary, the findings indicate its importance from the opposite perspective. Respondents repeatedly mentioned the lack of organizational support, including the absence of regular psychological counseling mechanisms, professional psychological consultation channels, and systematic experience-sharing platforms. This suggests that organizational-level coping is not absent in principle, but its actual provision remains clearly insufficient. Therefore, organizational-level coping can be understood as a systematic arrangement that buffers referees’ pressure through institutionalized resource provision. It may include establishing psychological support mechanisms before, during, and after matches; improving mentoring and stratified training systems; promoting institutionalized post-match review and case-based feedback; optimizing evaluation and promotion mechanisms; and building stable platforms for peer communication and experience sharing. From the perspective of model relationships, organizational coping can not only directly reduce the accumulation of relational pressure and decision-making pressure but also indirectly strengthen individual adjustment by improving referees’ professional competence, psychological resilience, and recovery ability. Therefore, this study argues that the individualized nature of current coping strategies is both an empirical fact and a result of insufficient organizational support. In the future, organizational-level coping should be incorporated into referee stress intervention and training systems to promote a shift from individual independent coping to systematic coping supported by organizations.

Student basketball referees may experience psychological fluctuations and somatic stress responses under pressure. Athletes and officials commonly use breathing-based relaxation techniques to regulate excessive arousal. Controlled studies indicate that the 4–7–8 breathing technique may reduce state anxiety and influence autonomic and cardiovascular indicators, including heart-rate variability, heart rate, and blood pressure (Aktaş and İlgin, 2023; Goldsmith and Williams, 1992; Vierra et al., 2022). Although these findings were not obtained from basketball referees, they provide a preliminary basis for examining breathing-based interventions in officiating contexts.

From a growth-mindset perspective, individuals who believe that abilities can be developed through effort and experience are more likely to persist and seek effective strategies following failure (Dweck and Yeager, 2019). This growth-oriented cognition may serve as a protective factor in stress regulation. Stress-response regulation focuses on optimizing responses to facilitate goal attainment; when individuals regard stress as potentially beneficial, they may be more likely to channel it toward adaptive performance rather than merely attempting to suppress it (Crum et al., 2020). Research on coping styles has distinguished approach-oriented from avoidant responses to acute stress (Anshel et al., 2000). In addition, imagery, relaxation breathing, self-talk, and communication-focused strategies have been identified as useful components of stress management for sport officials (Voight, 2009).

### 4.3 Mechanisms of referee pressure on decision-making and psychological behavior

The officiating pressure experienced by college basketball referees does not follow a static stimulus–response pattern. Instead, it constitutes a dynamic, multilayered, and complex interactive system. The qualitative analysis in this study reveals that pressure influences referees’ cognitive, emotional, and physiological states, thereby affecting their decision-making behavior and psychological stability. This mechanism appears to exhibit two distinct characteristics: bidirectionality and dynamic cyclicality.

#### 4.3.1 Pathways through which pressure affects officiating performance

Performance through two pathways: increased cognitive load and decision-making biases.

First, in high-level competitions, multiple stressors, such as crowd noise, coach protests, and critical scores, compete for referees’ limited cognitive resources and significantly increase cognitive load. When an individual’s information-processing capacity becomes overloaded, problems such as distracted attention, reduced visual search efficiency, and slowed information retrieval may occur (Helsen and Bultynck, 2004). For referees, this may manifest as difficulty tracking all critical details during rapid offensive-defensive transitions and a reduced ability to detect subtle fouls, thereby increasing the risk of missed calls and incorrect decisions. Multiple interviewees noted that in the later stages of matches, declining physical stamina and accumulated fatigue made it difficult to maintain concentration, which consequently reduced the sharpness and accuracy of officiating decisions.

Second, pressure may induce systematic decision-making biases. On the one hand, under self-doubt or external scrutiny, referees may develop a tendency to avoid making calls. That is, they may hesitate when judging borderline or 50/50 actions in order to prevent controversy, thereby missing the optimal timing for decision-making. On the other hand, to compensate for previous errors or resist external pressure, referees may display fluctuating decision-making standards, which undermines consistency during matches. In addition, under sustained pressure from the home crowd or assertive coaches, referees’ decisions may be unconsciously influenced, resulting in subtle but unfair tendencies (Yao, 2015).

#### 4.3.2 Psychological and behavioral responses triggered by pressure

Pressure not only affects cognitive decision-making but also triggers a chain of psychological and behavioral responses.

Psychologically, pressure first induces emotional fluctuations. After officiating an important match for the first time or making a decision-making error, referees often report feelings of tension, anxiety, self-blame, and even guilt. If these emotions are not effectively managed, they may further erode referees’ confidence in decision-making and their professional identity. Some interviewees even reported that they began to doubt their suitability for refereeing.

Behaviorally, stress responses may manifest as somatization and avoidance tendencies. Physiologically, they may appear as increased heart rate, sweating, muscle tension, and other typical stress responses. In behavioral terms, some young referees may subconsciously reduce their movement and positioning, attempting to minimize their presence during officiating by “disappearing” in order to avoid becoming the focus of controversy. This passive officiating approach may paradoxically increase the likelihood of decision-making errors due to poor observation, thereby creating a vicious cycle.

#### 4.3.3 Regulatory effects of individuals and environment

The ultimate impact of pressure is moderated by both individual characteristics and environmental factors. At the individual level, referees’ professional competence and psychological resilience serve as key moderating variables. Nie Yu (Nie and Wang, 2023) suggests that building strong self-confidence, cultivating willpower, and improving emotional self-regulation are effective strategies for alleviating officiating pressure. Solid theoretical knowledge and extensive practical officiating experience provide an important foundation for accurate decisions, while strong psychological resilience helps referees maintain composure when facing criticism.

At the environmental level, support and collaboration within the refereeing team play a crucial buffering role. Communication and coordination among referees are particularly important, as a clear division of responsibilities and close cooperation can effectively reduce individual psychological pressure. However, systematic psychological support within current referee training systems remains inadequate. This study also reveals a general lack of organizational-level support, such as systematic psychological counseling and professional post-match debriefing mechanisms. As a result, college basketball referees often have to rely more heavily on their own limited resources to cope with pressure, which may increase the risk of a vicious cycle.

In summary, officiating pressure among college basketball referees influences their performance and psychological responses through cognitive and emotional mediating variables, ultimately leading to either growth or attrition within a dynamic cycle. This complex mechanism suggests that interventions should not focus only on reducing pressure sources. Instead, they should also cultivate referees’ positive attitudes toward stress and systematically strengthen their personal resources and social support systems.

#### 4.3.4 Development trajectory explanation

It should also be noted that the nature of officiating stress may differ across referees at different stages of experience. Although less experienced referees in this sample tended to report stronger overall pressure, the data also suggest—tentatively—that the focus of pressure may shift with accumulating experience. For novice or less experienced referees, pressure appeared to center more on technical competence and immediate task execution, including rule mastery, judgment accuracy, consistency of standards, positioning, and fear of making mistakes. For more experienced referees, pressure seemed to relate more to organizational and reputational concerns, such as performance evaluation, promotion opportunities, expectations in critical matches, public scrutiny, and the need to maintain consistently high standards across games. However, these observations are derived from a cross-sectional sample and reflect participants’ retrospective comparisons rather than actual developmental trajectories. They should therefore be interpreted as suggestive patterns that generate hypotheses for future longitudinal research, rather than as established developmental findings. We have noted this limitation explicitly in Section 5 (Conclusions and Research Limitations). It is important to clarify that the dual-cycle model presented here is derived from participants’ retrospective accounts of their experiences with officiating pressure. The relationships between pressure, coping, and outcomes described by participants suggest recurrent patterns that may be interpreted as cyclical in nature. However, given the cross-sectional design of this study, these patterns should be understood as perceived dynamics rather than causally established mechanisms. The terms “pressure-learning-growth” and “pressure-error-escalating pressure” are used to summarize participants’ narrative trajectories, not to assert causal directionality.

A further question is what determines whether referees, after experiencing a stressful event, enter a positive cycle of pressure, learning, and growth or become trapped in a negative cycle of pressure, error, and escalating pressure. Based on the coding results and theoretical integration, this study identifies three key transition mechanisms.

First, self-efficacy constitutes the core basis for referees’ secondary appraisal. Referees with higher self-efficacy are more likely to appraise stressful events as controllable and remediable. As a result, they tend to adopt active coping strategies, such as calm reflection and active communication. Successful coping can further reinforce self-efficacy and form a resource-gain spiral. In contrast, referees with lower self-efficacy are more likely to appraise the same events as uncontrollable or as exposing their inadequacies. This may trigger self-blame and avoidance, thereby forming a resource-loss spiral. Second, stress mindset influences how referees make meaning of stressful situations. Referees with a stress-enhancing mindset are more inclined to reappraise high-pressure situations as challenges, which may stimulate concentration and performance potential. By contrast, referees with a stress-debilitating mindset are more likely to reappraise these situations as threats, which may trigger anxiety and hesitation. This cognitive reappraisal process directly shapes emotional responses and behavioral choices after stressful events. Third, social support and organizational support constitute the buffering mechanism of referees’ external resource environment. When referees receive immediate peer communication, mentoring-based debriefing, or institutional support after a stressful event, such as post-match psychological counseling or objective assessment of controversial decisions, their stress response is more likely to shift from internal depletion to externally supported recovery. This may interrupt the self-reinforcing negative spiral. When such support is absent, referees are forced to cope with stress in isolation, making them more susceptible to self-blame and hesitation in a self-reinforcing loop.

These three mechanisms interact to shape referees’ recovery trajectories after stressful events and constitute the key transition point between positive and negative cycles. This also suggests that effective interventions should not focus solely on reducing stressors. Instead, they should simultaneously enhance referees’ self-efficacy, cultivate stress-enhancing mindsets, and build stable external support systems. In this way, interventions can guide referees toward a positive developmental trajectory during the critical window between a stressful event and the next officiating decision.

### 4.4 The dual nature of stress effects

The theoretical integration presented in this section should be understood as a theory-informed interpretive effort rather than as a set of claims directly validated by the data. The transactional model of stress and coping (Lazarus and Folkman, 1984) and Conservation of Resources theory (Hobfoll, 1989) are used as overarching frameworks to organize and interpret patterns identified in the interview data. Stress mindset theory (Crum et al., 2013) is used to describe differences in how participants appraised pressure. These theories function as interpretive lenses rather than as empirically validated outputs of the grounded theory analysis. The dual-cycle model developed in this study should therefore be viewed as an empirically grounded conceptual account of participants’ experiences that is interpreted in relation to existing theory.

Stress has often been conceptualized primarily as a negative influence associated with adverse psychological and behavioral outcomes (Schraml, 2013; Dell et al., 2016). In high-intensity task situations, ineffective regulation of emotional and behavioral responses may contribute to anxiety, performance instability, and withdrawal intentions. However, the consequences of stress depend partly on how demands are appraised and on the coping resources available to the individual.

A key finding of this study is that officiating pressure is not inherently detrimental. Its ultimate impact appears to depend on the dynamic interaction between pressure intensity and individual coping abilities, potentially forming distinctly different cycles of influence.

#### 4.4.1 Vicious cycle: pressure-mistake-escalating pressure

When referees perceive pressure as an uncontrollable threat, they may easily fall into a vicious cycle of pressure, error, and escalating pressure (Crum et al., 2013). In this pattern, an initial pressure event, such as a controversial decision, triggers anxiety and self-doubt, impairs cognitive functioning, and increases the likelihood of officiating errors. Subsequent audience boos, coach protests, or self-criticism may further intensify psychological pressure, leading to increased hesitation or decision-making lapses in subsequent decisions. This cycle may ultimately result in professional burnout or even withdrawal from refereeing.

#### 4.4.2 Positive cycle: pressure–learning–growth

If referees interpret pressure as a manageable challenge, they may enter a positive cycle of pressure, learning, and growth (Jamieson et al., 2010). Moderate pressure can stimulate a sense of responsibility and concentration, enabling referees to mobilize their physical and mental resources to make critical decisions successfully under intense conditions. Such successful officiating experiences generate a strong sense of accomplishment and positive feedback, thereby significantly enhancing self-efficacy (Bandura, 1997). Referees with a growth mindset are better able to reflect on and learn from each high-pressure experience, viewing stress as a process through which they can refine their officiating skills (Dweck and Yeager, 2019). This approach enables simultaneous improvement in both professional competence and psychological resilience.

These dual pathways are consistent with contemporary research on stress mindset. A stress-debilitating mindset may amplify negative cognitive appraisals and physiological responses to pressure, thereby magnifying its adverse effects. Crum’s stress mindset theory suggests that individuals’ fundamental cognitive appraisal of stress—whether stress is perceived as enhancing or debilitating—shapes the direction of their physiological, emotional, and behavioral responses (Crum et al., 2013) Individuals who believe that stress is harmful are more likely to activate defensive stress responses and fall into cycles of tension and avoidance. Conversely, those with a stress-enhancing mindset, who view stress as conducive to growth, learning, and improvement, are more likely to engage in proactive coping behaviors and achieve positive outcomes.

A growing body of research emphasizes the dual nature of stress. With appropriate regulation and cognitive reframing, stress can become not an obstacle but an important driving force that motivates individuals, enhances adaptability, and promotes professional development (Jamieson et al., 2010; Dienstbier, 1989). This is particularly evident in the career development of student basketball referees. Positive pressure often stems from the high standards and expectations embedded in competitive environments. It can strengthen referees’ sense of responsibility and professional mission, prompting them to maintain greater concentration and make more accurate decisions during officiating tasks. In the interviews conducted in this study, multiple student basketball referees reported that although external pressure in high-level competitions initially caused anxiety, it also motivated them to continuously deepen their understanding of rules and improve their on-site decision-making abilities. Positive feedback after successful critical decisions often becomes an important source of self-efficacy and professional confidence. This experience of drawing energy from pressure is closely consistent with the theory of the stress-enhancing mindset. Under this mindset, individuals can redefine pressure as a challenge rather than a threat and adopt more proactive coping strategies, such as advance preparation, emotional regulation, and on-site reappraisal.

Crum (Crum et al., 2023) further demonstrated through intervention studies that individuals can change their perceptions of stress by watching brief educational videos or engaging in reflective tasks, which may lead to improved performance and better mental health in work and academic settings. Under a positive stress mindset, student basketball referees are more likely to view high-pressure matches as opportunities to demonstrate their professional competence rather than as sources of catastrophic risk. These referees typically show stronger problem-solving abilities, better emotional regulation, greater resilience to setbacks, and higher consistency in officiating. However, the negative effects of stress cannot be overlooked. In the interviews, multiple student basketball referees reported significant psychological fluctuations, including anxiety, self-blame, and guilt, after experiencing disputes during matches, public skepticism, or consecutive officiating errors. Some even expressed doubts about their suitability for refereeing. If these negative experiences are not properly addressed, they may develop into professional burnout and weaken referees’ commitment to continuing officiating. In particular, without systematic support or cognitive regulation skills, prolonged exposure to high-pressure environments may trigger chronic stress responses. This may ultimately lead to marginalization within the refereeing community or voluntary withdrawal from refereeing.

In summary, the pressure experienced by college basketball referees is highly dynamic and situation-specific. Its effects are shaped by the interplay of multiple factors, including stress mindset, self-efficacy, social support systems, and coping strategies (Dweck and Yeager, 2019; Crum et al., 2023). A scientific understanding and effective use of pressure not only affect the mental health and officiating quality of individual referees but also contribute to the professionalization and stable development of the refereeing workforce. Therefore, universities and relevant organizations should incorporate intervention pathways into referee training, including stress perception restructuring, mindfulness training, contextualized simulation exercises, and post-match debriefing or psychological counseling. These approaches can help student basketball referees adapt to pressure psychologically, transform pressure cognitively, and optimize performance behaviorally.

It should be noted that although the concept of stress mindset was informed by the work of Crum and colleagues during the discussion stage, its empirical basis emerged inductively from the interview materials. During open coding, participants’ statements, such as “pressure helps me focus more” and “the more pressure there is, the easier it is to make mistakes,” reflected different implicit beliefs about the nature of pressure. During axial and selective coding, these statements were progressively integrated into the category of stress appraisal tendency, which ultimately became a key moderating mechanism in the dual-cycle model distinguishing between growth cycles and loss cycles. In other words, stress mindset in this study was not imposed as an external theoretical framework after data analysis. Rather, it was developed through conceptual integration between the inductively emerged category of stress appraisal tendency and existing theory. Placing these findings within a broader theoretical context reveals several extensions and refinements that this study contributes to existing stress theories.

First, the dual-cycle model constructed in this study provides a mechanism-based extension of the transactional model proposed by Lazarus and Folkman (Lazarus and Folkman, 1984). The transactional model emphasizes that stress results from cognitive appraisal and coping, but it does not fully explain how stress outcomes may reciprocally shape future stress processes through cumulative cycles. This study reveals that referees’ coping outcomes after stressful events, whether successful or unsuccessful, can reciprocally influence their self-efficacy, stress mindset, and access to external resources. These changes may further alter the appraisal starting point and coping capacity for the next stressful event. This cumulative cyclical mechanism extends the stress process from a single event to a dynamic developmental trajectory, thereby offering theoretical value for understanding stress adaptation and psychological resilience.

Second, this study’s elucidation of organizational-level pressure provides a contextual supplement to the concept of resource threat in COR theory (Hobfoll, 1989). Among college basketball referees, organizational-level pressures, such as evaluation scoring, promotion competition, and task arrangements, do not merely constitute resource deficits. Rather, they represent institutional resource threats. Referees must cope not only with the immediate pressure of match situations but also with the sustained expectation of resource depletion generated by the evaluation system. This finding suggests that stress interventions should not focus solely on individual-level resource replenishment. Instead, they should also examine whether institutional arrangements themselves function as stressors.

Third, the integrated framework of “personal factors—external support—cycle direction” proposed in this study incorporates self-efficacy theory (Bandura, 1997), stress mindset theory (Crum et al., 2020; Crum et al., 2013; Crum et al., 2023) and COR theory (Hobfoll, 1989) into a unified explanatory system: personal factors (self-efficacy, professional competence) determine the abundance of referees’ resource pools; stress mindset determines the direction of referees’ cognitive appraisal of stressful situations (challenge or threat); external support (social support, organizational support) provides the macro-environment for resource replenishment or resource threat; and the interaction of these three jointly determines whether referees enter a resource gain spiral or a resource loss spiral. This integrated framework provides a testable hypothetical pathway for future research and offers multi-level targets for stress intervention design.

Through this theoretical dialogue and integration, this study not only empirically reveals the sources of officiating stress and the coping strategies used by college basketball referees, but also theoretically extends the understanding of the dynamic mechanisms linking stress, coping, and development. In doing so, it provides a more process-oriented and systematic analytical framework for research on the psychology of sports officials.

From a broader theoretical perspective, the model of referee stress constructed in this study is highly consistent with Hobfoll’s Conservation of Resources (COR) theory (Hobfoll, 1989) COR theory posits that individuals strive to acquire, retain, and protect their resources. Stress occurs when resources are threatened, actually lost, or when resource investment does not produce expected returns. The abundance of resources also determines individuals’ adaptive capacity and coping outcomes in stressful situations.

In the model developed in this study, personal factors, including physical fitness, psychological resilience, and professional competence, constitute referees’ internal resource pool for coping with stress. Relational and organizational support constitute the external resource environment. The dual-cycle model corresponds to the dynamics of resource-gain and resource-loss spirals. Specifically, when referees possess abundant internal resources and receive stable external support, they are more likely to appraise high-pressure matches as manageable challenges. Through successful coping, they can further replenish resources, such as increased confidence and accumulated experience, thereby forming a resource-gain spiral. Conversely, when referees lack sufficient internal resources and external support is absent, pressure is more likely to be appraised as an uncontrollable threat. Failed coping may further deplete resources and form a resource-loss spiral.

Within this framework, stress mindset can be understood as a resource-investment decision tendency. Referees with a stress-enhancing mindset are more inclined to mobilize resources under pressure in order to obtain growth-related returns, whereas those with a stress-debilitating mindset are more likely to avoid resource investment in order to prevent further resource loss. Introducing COR theory not only integrates personal factors, social support, and organizational-level pressure into a unified resource system, but also provides a micro-theoretical foundation for understanding the formation mechanisms of the dual cycles.

It should be noted that although the concept of stress mindset was informed by the research of Crum and colleagues at the discussion stage, its empirical basis emerged inductively from the interview materials. During open coding, statements from participants such as “pressure helps me focus more” or “the more pressure, the easier it is to make mistakes” reflected referees’ different implicit beliefs about the nature of pressure; during axial and selective coding, these statements were progressively integrated as “stress appraisal tendency” and ultimately constituted a key moderating node in the dual-cycle model distinguishing between “growth cycles” and “loss cycles.” To be precise, what emerged inductively was the empirical pattern of differing pressure beliefs; the theoretical label “stress mindset” was introduced afterwards to facilitate interpretation and theoretical dialogue.

To provide further empirical grounding, representative quotations from participants are presented below. One referee stated: “I think pressure is a good thing; it means the game is important, and I consciously make myself more focused.” Another said: “When the pressure is high, I feel like something is going to go wrong; the more I worry about making a mistake, the easier it is to make one.” These contrasting statements, along with similar expressions from other participants, were coded under the category “pressure appraisal tendency” during open and axial coding. The theoretical concept of “stress mindset” (Crum et al., 2013) was then introduced at the interpretation stage to provide a recognized label and theoretical framework for this empirical pattern. Thus, the empirical phenomenon emerged from the data, while its theoretical naming and articulation drew on existing literature.

## 5 Conclusions and research limitations

This study offers a grounded account of how officiating stress is experienced and managed by male student basketball referees in China. The findings identify five interrelated sources of stress spanning internal resources, interpersonal and organizational relations, officiating decisions, match conditions, and tournament context. These pressures converged most directly in the decision-making process: situational and relational demands intensified decisional strain, whereas personal resources such as physical fitness, psychological resilience, and officiating competence shaped stress appraisal. Across participants’ accounts, these pressures were associated with somatic, cognitive, and emotional responses and with changes in perceived officiating stability and confidence.

To manage these demands, referees relied primarily on self-regulation, proximal social support, and competence-building strategies, including pre-match mental preparation, breathing regulation, peer communication, rule learning, physical training, and post-match review. The findings also suggest a dual pattern in how pressure was experienced: when pressure was appraised as manageable and accompanied by sufficient personal and social resources, it was linked to concentration, learning, and professional growth; when it was experienced as overwhelming, it was associated with hesitation, perceived errors, and escalating strain. These results underscore the need for referee development systems that integrate psychological skills training, structured mentoring, and organizational support rather than leaving student referees to rely mainly on individual coping.

Several limitations should be acknowledged. The sample was limited to male National Level-I student basketball referees in China, which constrains transferability to other referee populations, sports, and cultural settings. The study relied on retrospective, cross-sectional qualitative accounts rather than longitudinal observation, and it did not include objective psychological or physiological indicators; therefore, the proposed “pressure-learning-growth” and “pressure-error-escalating pressure” patterns should be interpreted as perceived narrative processes rather than causal or developmental trajectories. In addition, the insider positioning of parts of the research team, including prior professional familiarity with some participants, may have shaped data generation and interpretation despite reflexive safeguards, some interviews were relatively short, and recollections of stressful officiating episodes were inevitably subject to recall bias. Future research should test and refine the model using more diverse samples, longitudinal or mixed-method designs, and complementary psychological or physiological measures.

## 6 Suggestions and outlooks

### 6.1 Practical implications

The findings of this study offer practical implications for sports federations, university athletic departments, and referee associations seeking to develop more effective support systems for college basketball referees. Rather than treating officiating pressure merely as a problem to be reduced, support systems should help referees transform pressure into a structured process of adaptation, learning, and professional growth. At the individual level, the findings highlight the need for targeted psychological skills training and competence-development programs. These programs may include mindfulness-based pre-match preparation, breathing regulation, self-talk training, and stress mindset education. They should also include structured learning modules on rule mastery, decision-making consistency, positioning, and post-match reflection. Such interventions may enhance referees’ self-efficacy, emotional stability, and task performance under pressure.

At the interpersonal level, the prominence of relational pressure and coordination demands underscores the importance of structured peer-support systems. Sports organizations may implement pre-match communication routines, coordination training during officiating, post-match peer debriefing, and mentoring schemes in which experienced referees, technical representatives, or referee instructors support less experienced officials. These approaches may reduce feelings of isolation, strengthen collaborative confidence, and improve referees’ ability to manage complex match situations. At the organizational level, the findings point to the need for more formalized and sustained institutional support. This may include access to psychological counseling services, systematic post-match review and recovery procedures, transparent and equitable evaluation and promotion mechanisms, and continuous development pathways for early-career referees. Such institutional arrangements are important for strengthening long-term psychological resilience and supporting referees’ professional development. Overall, the proposed model provides a practical framework for designing integrated interventions that align psychological preparation, peer support, skill development, and organizational policy. In this sense, referee development should move beyond fragmented individual coping and toward a more systematic support structure that enables referees to adapt to pressure, improve decision-making, and sustain professional growth.

### 6.2 Policy recommendations

At the policy level, the findings suggest the need for a more institutionalized referee development pathway led by sports federations, university athletic authorities, and relevant governing bodies. First, psychological support should be formally embedded into referee education and certification systems rather than treated as an *ad hoc* response to crisis situations. This may include mandatory modules on stress recognition, emotional regulation, pre-match psychological preparation, coping during officiating, and post-match recovery within referee training curricula. Second, federations and referee associations should consider establishing structured mentorship programs. In these programs, experienced referees, technical representatives, or referee instructors could provide continuous developmental guidance to younger officials, especially in relation to critical-match preparation, controversial decisions, confidence rebuilding, and career progression. Third, evaluation and promotion systems should be improved to reduce excessive reliance on single-match performance ratings. Greater attention should be given to transparency, developmental feedback, and fairness in advancement pathways. Such changes may reduce organizational-level pressure and help referees interpret evaluation not only as a source of threat but also as a mechanism for learning and improvement. Fourth, policy actors may create regularized post-match review systems, shared officiating case databases, and regional exchange platforms. These mechanisms would allow referee development to be supported by collective learning rather than fragmented individual experience alone. Taken together, these recommendations point to a four-part institutional framework for referee development: psychological support, mentorship, stress education, and developmental evaluation. Such a framework may help transform current support gaps into a more sustainable and growth-oriented system. It would not only address the organizational deficiencies identified in this study but also reduce the likelihood that referees become trapped in a negative cycle of pressure, error, and escalating pressure.

## Data Availability

The original contributions presented in the study are included in the article/Supplementary material, further inquiries can be directed to the corresponding author.
