# Supplementary material for: Officiating stress and coping strategies among male student basketball referees in China: a procedural grounded theory study
Source: Front Psychol. 2026 Jun 26;17:1794393. doi: 10.3389/fpsyg.2026.1794393 (PMC13349394; doi:10.3389/fpsyg.2026.1794393)
Supplement: Supplementary file 1 [file Data_Sheet_1.PDF]

Supplementary Document 1

Semi-Structured Interview Guide

Officiating Stress and Coping among Male Student Basketball Referees in China

1. Opening Script

Thank you for taking the time to participate in this interview. The purpose of this study is to gain an in-depth understanding of the sources and consequences of stress experienced by student basketball referees during officiating. The interview is expected to last approximately 30 minutes. Your identity and responses will be kept confidential and used solely for academic research. With your permission, the interview will be audio-recorded in full.

2. Participant Background and Officiating Experience

1. Age
2. Gender
3. Highest level of education completed or currently pursued
4. Year in which National Level-I referee certification was obtained
5. Total years of officiating experience
6. Approximate number of national-level competition games officiated
7. Which major national competitions have you officiated? Which match left the strongest impression on you, and why?

3. Main Interview Questions

1. During your previous experiences officiating national-level competitions, have you encountered any situations that made you feel particularly stressed? What do you consider to be the main sources of officiating stress for student basketball referees?
2. When you experience officiating stress, what physical or somatic reactions do you notice? What practical or physical strategies do you use to manage these reactions?
3. How do you usually cope with officiating stress?
4. Are there any stressors or stress reactions that remain difficult to manage even after you have used coping strategies? Why do you think these difficulties persist?
5. How does the intensity of competition, such as frequent physical contact or rapid offensive-defensive transitions, affect your officiating? Does it increase your stress? Please describe how.
6. How do you feel when making critical decisions, such as a foul call that may influence the outcome of a match or a controversial call late in the game?
7. Beyond your own efforts, what support, if any, have your university, referee association, or other organizations provided to help referees manage officiating stress?

4. Interviewer Guidance

The interviewer should use the questions flexibly and may ask neutral follow-up questions to clarify participants' accounts, obtain concrete examples, and explore emerging issues. Leading, evaluative, or judgmental responses should be avoided.

5. Closing Script

Thank you again for your time and valuable contribution. The information you have provided is highly important to this study.
