# Supplementary material for: Officiating stress and coping strategies among male student basketball referees in China: a procedural grounded theory study
Source: Front Psychol. 2026 Jun 26;17:1794393. doi: 10.3389/fpsyg.2026.1794393 (PMC13349394; doi:10.3389/fpsyg.2026.1794393)
Supplement: Supplementary file 2 [file Data_Sheet_2.PDF]

Supplementary Document 2. Trajectories of the emergence of concepts and categories among the participants

Primary Category	Corresponding Category	Includes the concept	Original Citation
Personal factors	Physical factors	Action execution, physiological load, mental fatigue	After playing the entire match, my legs became completely weak; in the final minutes, I simply couldn't keep up with the ball's speed, leading to frequent misjudgments.
	Psychological factors	Emotional regulation ability, psychological sensitivity, psychological resilience, self-efficacy	Psychological resilience is crucial for referees. An outstanding referee must quickly regain composure after making a controversial call and avoid allowing emotions to interfere with subsequent officiating decisions, which requires long-term, systematic psychological training.
	Professional competence	Individual officiating competence, communication and coordination skills, mastery of rules, on-the-spot adaptability and decision-making ability, and accumulated officiating experience.	The accumulation of refereeing experience is irreplaceable. While young referees often demonstrate precise technical skills, their ability to make comprehensive judgments in chaotic situations and their communication skills with athletes and coaches require time and extensive practical experience to develop.

Relationship stress	Interpersonal stress	Interpersonal pressure among referees, interpersonal interaction pressure within the organizing committee	There exists an underlying interpersonal tension within the refereeing team, where seniority, factions, and personal interests all influence relationships among members. These external factors can sometimes manifest on the field, affecting the referees' performance.
	Social pressure	Media coverage, peer reviews, online public opinion	Media coverage of referees is often selective; any controversial call is invariably amplified. Under prolonged public scrutiny, referees' professional dignity suffers significant erosion.
	Pressure on Referee Coordination	Differences in experience pressure, lack of coordination, and variations in officiating styles	The overall coordination of the refereeing team is crucial to ensuring high-quality officiating; differences in style, experience gaps, or inadequate communication mechanisms can lead to inconsistent penalty standards, necessitating thorough collaboration before the match.
	Organizational relationship pressure	Evaluation scoring pressure, promotion pressure, seasonal task pressure,	There are only a limited number of promotion spots, and everyone is competing

		insufficient organizational support pressure	fiercely. You know someone is watching every single one of your performances — this pressure lasts longer than that on the field itself.
Pressure of Refereeing Decisions	Pressure on Referee Decision-Making	Conflict between fairness and subjective judgment in officiating, pressure to maintain consistent standards in officiating	A persistent tension exists between the fairness of officiating and the inherent cognitive limitations of referees. How to achieve the greatest possible objectivity and fairness, given the inevitability of subjective judgment, remains a lifelong challenge for every referee.
	Pressure of Referee Decisions	The intensity of the game's pace, the comprehensive adjudication of multi-factor conflict scenarios, the complexity and stealthiness of foul actions, and the disconnect between rule comprehension and on-field situations.	Modern basketball's offensive and defensive intensity, along with its tactical complexity, far exceed the scenarios originally envisioned when the rules were established. Many real-world conflict situations cannot be precisely covered by the rulebook, requiring referees to exercise holistic judgment that transcends legal provisions.
	Timing of Penalty Calls	Ruling too hastily: Rash, erroneous judgment Ruling too slowly: Missing the opportunity	Hesitation is a major pitfall. I've witnessed referees taking two seconds to make a call, by which time

			the players had already engaged in combat, exacerbating the chaos on the field. Once the critical moment is missed, even the most accurate decision appears ineffective.
	Accuracy of Refereeing Decisions	Accuracy of critical calls, pressure from missed or incorrect calls, and accurate calls boosting confidence.	Recurrent misjudgments erode confidence and foster refereeing anxiety, whereas high-quality accurate calls provide positive reinforcement, helping referees establish a stable psychological foundation for officiating.
Competition pressure	Competition environment pressure	Venue environment induces stress, multiple pressures compounding on the field, unexpected situations during play, spectator atmosphere exerting pressure, coaching staff applying pressure, athletes pressuring themselves.	Lighting conditions, noise levels, audience emotions, coaching interventions, and athlete pressure collectively create a highly stressful refereeing environment. Referees must possess the ability to maintain focus and composure amidst these multiple stimuli.
	Competitive pressure on the field	Competition pressure, match intensity, scoreboard pressure, critical calls, player skill level	When facing top-tier teams, the pace of play is extremely fast and physicality intense; some technical fouls are concealed within seamless teamwork

			and hard to spot. The more skilled the players, the greater the challenge for referees — only those who truly understand the game can effectively manage them.
Competition and Match Pressure	Crucial Matches	Championship showdown, powerhouse clash, rivalry showdown, knockout stage advancement, home team's crucial match	The refereeing pressure in finals or high-stakes matches is unique: attention from both teams, club management, media, and fans reaches its peak simultaneously, requiring referees to handle exceptionally demanding technical and tactical challenges.
	Competition Level	The heightened standards for high-level competitions create pressure, and the outcomes of such competitions carry significant implications.	The higher the competition level, the lower the fault tolerance rate. A similar missed call might go unnoticed in lower-tier events, but at national-level competitions, it appears in various analysis videos the very next day.
