# Supplementary material for: Officiating stress and coping strategies among male student basketball referees in China: a procedural grounded theory study
Source: Front Psychol. 2026 Jun 26;17:1794393. doi: 10.3389/fpsyg.2026.1794393 (PMC13349394; doi:10.3389/fpsyg.2026.1794393)
Supplement: Supplementary file 5 [file Data_Sheet_5.PDF]

Supplementary Document 5. Participant Characteristics

N = 28 male student basketball referees; age: 23.43 ± 2.33 years; officiating experience: 4.29 ± 2.14 years; education: Undergraduate = 14, Master's = 13, PhD = 1; eligible for national-level promotion: 14 yes, 14 no.

ID	Gender	Age	Education	Experience (years)	National-level games	Eligible for national-level promotion	ID	Gender	Age	Education	Experience (years)	National-level games	Eligible for national-level promotion
R1	Male	26	PhD	8	4	Yes	R15	Male	23	Master's	3	1	No
R2	Male	22	Undergraduate	4	2	Yes	R16	Male	25	Master's	4	1	No
R3	Male	23	Master's	4	2	Yes	R17	Male	22	Undergraduate	3	1	No
R4	Male	25	Master's	7	3	Yes	R18	Male	22	Undergraduate	3	1	No
R5	Male	27	Master's	6	5	Yes	R19	Male	21	Undergraduate	3	1	No
R6	Male	24	Master's	3	1	No	R20	Male	21	Undergraduate	3	1	No
R7	Male	23	Undergraduate	3	1	Yes	R21	Male	27	Master's	8	1	No
R8	Male	25	Master's	5	3	Yes	R22	Male	26	Undergraduate	5	1	No
R9	Male	24	Master's	2	1	No	R23	Male	25	Master's	7	1	No
R10	Male	29	Master's	10	2	Yes	R24	Male	23	Undergraduate	2	1	No
R11	Male	24	Master's	6	3	Yes	R25	Male	21	Undergraduate	4	1	No
R12	Male	24	Master's	5	1	Yes	R26	Male	21	Undergraduate	2	1	No
R13	Male	22	Undergraduate	3	1	Yes	R27	Male	20	Undergraduate	3	1	Yes
R14	Male	22	Undergraduate	2	1	Yes	R28	Male	19	Undergraduate	2	1	Yes

Note. The table presents the final analytic sample of 28 male student basketball referees. All participants were certified National Level-I Basketball Referees recognized by the Chinese Basketball Association. National-level games refer to national youth or student basketball competitions officiated by the participants.
